# Efficacy of chitosan-based nanoparticle vaccine administered to broiler birds challenged with *Salmonella*

**DOI:** 10.1371/journal.pone.0231998

**Published:** 2020-04-24

**Authors:** Keila Y. Acevedo-Villanueva, Bailey Lester, Sankar Renu, Yi Han, Revathi Shanmugasundaram, Renukaradhya Gourapura, Ramesh Selvaraj

**Affiliations:** 1 Department of Poultry Sciences, University of Georgia, Athens, Georgia, United States of America; 2 Food Animal Research Program, Ohio Agricultural Research and Development Center, The Ohio State University, Wooster, Ohio, United States of America; 3 Department Of Veterinary Preventive Medicine, College Of Veterinary Medicine, The Ohio State University, Columbus, Ohio, United States of America; Midwestern University, UNITED STATES

## Abstract

Two experiments were conducted to evaluate the immune response of broilers vaccinated with *Salmonella* chitosan-nanoparticle (CNP) vaccine and challenged with *Salmonella*. The *Salmonella* CNP vaccine was synthesized with *Salmonella* enterica outer membrane proteins (OMPs) and flagellin proteins. In Experiment I, birds were orally gavaged with PBS or 500, 1000, or 2000μg of CNP vaccine 1 and 7d-of-age. At 14d-of-age, birds were orally challenged with 1 X 10^5^ CFU/bird of live *S*. Enteritidis (SE). Macrophage-nitrite production 11d-post-challenge was higher (*P<*0.05) in the 500μg group when compared to the control. At d14 (8h-post-challenge), broilers vaccinated with 1000μg CNP had higher (*P<*0.05) serum anti-OMPs IgG and IgA and cloacal anti-OMP IgA amounts. At 11d-post-challenge, birds vaccinated with 1000μg CNP vaccine had greater (*P<*0.05) bile anti-OMP and anti-flagellin IgA amounts. At 11d-post-challenge, birds administered 1000μg CNP vaccine has increased (*P<*0.05) IL-1β and IL-10 mRNA in cecal tonsils. In Experiment II, birds were orally gavaged with PBS or 1000μg CNP or a live commercial vaccine at 1 and 7d-of-age. At 14d-of-age, birds were orally challenged with 1 X 10^5^ CFU/bird of live SE or *S*. Heidelberg (SH). Birds vaccinated with CNP showed higher (*P<*0.05) serum anti-OMPs IgG amounts at 8h-post-challenge. At 4d-post-SH challenge, birds vaccinated with CNP had higher (*P<*0.05) bile anti-flagellin IgA amounts. CNP decreased (*P<*0.05) anti-OMPs IgG levels in serum at 2d-post-SE challenge and 4d-post-SH or SE challenge. *Salmonella* Enteritidis loads in cecal content at 2d-post-challenge was decreased (*P<*0.05) by 65.9% in birds vaccinated with CNP, when compared to the control. Chitosan-nanovaccine had no adverse effects on bird’s production performance. In conclusion, 1000μg CNP vaccine can induce a specific immune response against *Salmonella* and has the potential to mitigate SE cecal colonization in broiler birds.

## Introduction

In the United States, there are over 40,000 reported cases of *Salmonella* infection in humans, and approximately 400 deaths are reported annually [1]. Consumption of contaminated poultry meat and egg products causes salmonellosis in humans [2]. Salmonellosis symptoms include stomach irritation accompanied by vomiting, diarrhea, high fever, and even death in humans, especially in immunocompromised individuals [3]. *Salmonella* enterica serovars Enteritidis (*S*. Enteritidis) and Heidelberg (*S*. Heidelberg) are among the most frequent serotypes recovered from humans each year [4]. *S*. Heidelberg has been isolated from poultry products in Brazil since 1962, while *S*. Enteritidis is a severe problem in poultry and public health since 1993 [5]. Upon ingestion, *Salmonella* will survive passage through the low-pH conditions of the stomach [6,7], stimulate macrophages, and evade killing by the host immune system [8]. Within a few hours, *Salmonella* can invade the intestinal tract and reach the liver and spleen, and *Salmonella* can colonize the ceca in chickens [9].

Vaccination is one of the most promising control strategies for the reduction of *Salmonella* in chickens [10]. Cross-protection can enhance the clearance of pathogens through the acquired immune response [10,11]. *Salmonella* serovars have conserved antigens and including conserved antigens in vaccines might induce cross-protection against multiple serovars [12]. Flagella [13] and outer membrane proteins (OMPs) [14] are conserved antigens among several *Salmonella* serovars that can induce an immune response in poultry.

Killed vaccines are preferred over live vaccines to control *Salmonella* infections of poultry because live vaccines can regain its virulence [15,16]. But killed vaccines have a disadvantage that killed vaccines need to be injected, which is time consuming and decreases the value of breast meat. Hence, oral administration of *Salmonella* vaccine is the preferred route [4] because oral administration mimics the natural infection and stimulates the mucosal and systemic immune responses [17]. However, oral killed *Salmonella* vaccines are not commercially available for broilers currently [18,19]. This article studies a nanoparticle based oral vaccine for *Salmonella* control in broilers.

Nanoparticle vaccines consist of a polymer coating that surrounds the vaccine antigen and protects the vaccine against chemical, enzymatic, and immunological degradation [20–22]. The prolonged survivability of the nanoparticles within the gastro intestinal tract (GIT) results in reducing the dosing frequency and the need for adjuvants [20], and facilitate the presentation of the vaccine antigens to mucosal immune cells [23]. Biodegradable chitosan nanoparticle (CNP) vaccines are ideal for delivering vaccine antigens through the oral route [23–25].

A *Salmonella* CNP vaccine was synthesized with a crude enriched OMPs and flagellin extracts from *S*. Enteritidis and surface-tagged with flagellin proteins. This research evaluated the protective effects of the synthesized CNP vaccine delivered through oral route in poultry by (1) Identifying the nanoparticle vaccine dose that can provide optimal immune response to *S*. Enteritidis infection, (2) Characterizing the CNP vaccine-induced anti-*Salmonella* OMPs and flagellar IgG and IgA specific antibodies in serum, cloacal swabs, and bile, (3) Identifying the effect of CNP vaccine on broilers performance parameters, pro- and anti-inflammatory cytokines, and (4) Evaluating the efficiency of the CNP vaccine on *S*. Enteritidis and *S*. Heidelberg loads in broiler birds challenged with *S*. Enteritidis and *S*. Heidelberg.

## Materials and methods

Two experiments were conducted to characterize the CNP vaccine-induced immune responses in broilers (Cobb-Vantress hatchery, Inc. Cleveland, GA, USA). CNP vaccine was synthesized at the Food Animal Health Research Program, The Ohio State University, USA as described earlier [26]. Experiment I identified the optimal dose of CNP vaccine that would induce a protective response against *S*. Enteritidis, and the Experiment II identified the cross protection of CNP vaccine against *S*. Heidelberg. All animal protocols were approved by the Institutional Animal Care and Use Committee at the University of Georgia. Birds were monitored at least once a day for bloody feces, lethargy, refusal to eat food, loss of body weight., diarrhea, and dehydration during experiment I and II.

### Preparation of *S*. Enteritidis CNP vaccines

OMPs and flagellin proteins were isolated from *S*. Enteritidis as described earlier [26]. CNP vaccine were prepared using the ionic gelation method where nanoparticles form by intramolecular and intermolecular crosslinking between the positively charged chitosan and the negatively charged sodium tripolyphosphate (TPP) [26]. Briefly, 1% (w/v) low molecular weight chitosan (Sigma, MO) solution was prepared by slowly dissolving chitosan in an aqueous solution of 4% acetic acid. The solution was sonicated, and the pH was adjusted to 4.3. The solution was filtered through a 0.44 μm syringe filter. Five milliliters of 1% chitosan solution was added to 5 mL of deionized water and incubated with 2.5 mg OMPs and flagellar proteins. Subsequently, 2.5 mL of 1% (w/v) TPP (Sigma Aldrich, St. Louis, MO) in 2.5 mL deionized water was added to the above solution under magnetic stirring at room temperature to form the nanoparticles. Afterwards, 2.5 mg of flagellin protein in PBS was added to the nanoparticles to surface conjugate the nanoparticles with flagellin proteins. The CNP vaccines were collected by centrifugation at 10,000 X g for 30 minutes, lyophilized and stored at -80°C until further use.

### Experiment I

Chicks in the experiment were confirmed to be *Salmonella* negative by streaking cloacal swabs on Xylose Lactose Tergitol^™^ 4 (XLT4) agar plates. A total of 14 one-day-old Cobb-500 chicks were distributed to one of the four treatment groups. The four treatment groups were control, 500μg, 1000μg, or 2000μg CNP vaccine groups. The control, 500μg, 1000μg, or 2000μg CNP vaccine groups had 5, 3, 3, and 3 birds on d1. At 1d of age, chickens were orally vaccinated with either 0.1 mL of PBS (Phosphate Buffered Saline; Control) or 500μg, 1000μg, or 2000μg CNP vaccine. A booster dose with the same amount of primary dose was given at d7. Birds were raised in individual battery cages and had access to *ad libitum* feed and water. At 14d of age, birds were challenged with 1 X 10^5^ CFU/bird of live *S*. Enteritidis orally.

At d1, 7, 14, 20, and 25 body weight and feed consumption were recorded, and body weight gain and feed consumption ratio was calculated. At d1, 7, 14 (8h post-challenge), 17, 20, 23, and 25 (11d-post-challenge) of age, serum was collected to analyze anti-OMPs and anti-flagellin IgG and IgA. At d17, 20, 23 and 25 (11d-post-challenge) of age, cloacal swabs were collected to analyze anti-OMPs and anti-flagellin IgA. At d14 (8h-post-challenge) and 25 (11d-post-challenge) of age, bile was collected to analyze anti-OMPs and anti-flagellin IgA. At d25 (11d-post-challenge) of age, cecal tonsils, liver, and spleen samples were analyzed for IL-1β, IL-4, IFNγ and IL-10 cytokine mRNA amounts, macrophage nitrite production and *Salmonella* loads in the cecal content.

### Macrophage-nitrite production in CNP vaccine administered birds

At d25 (11d-post-challenge) post-challenge, macrophages were collected from the spleen of one bird from each bird/treatment (n = 3) as described previously [27]. Single cell suspension of splenocytes were isolated using Histopaque (1.077 g/ml; Sigma Aldrich, St. Louis, MO). Briefly, 1 X 10^5^ splenocytes/well were plated in flat bottom 96 well plate (Greiner bio-one, NC) in triplicates. Cells were stimulated with 1 μg/mL *S*. Enteritidis lipopolysaccharide and incubated for 72 h at 37 °C in the presence of 5% CO_2_. Samples were centrifuged at 630 X g for 10 min at 4°C and 100 μL of the supernatants were removed. The nitrite content of the supernatant was determined using a sulfanilamide/N-(1-naphthyl) ethylenediamine dihydrochloride solution (Ricca Chemical Co., Arlington, TX) following the manufacturer’s instructions. Nitrite concentrations were determined from a standard curve with sodium nitrite standards [28].

### Antibody response in CNP-vaccine administered birds

At d25 (11d-post-challenge), serum, cloacal swabs, and bile samples were collected one bird/pen (n = 3) and analyzed for anti-*Salmonella* OMPs and -flagellin IgG and IgA antibody response using an enzyme-linked immunosorbent assay (ELISA) as described previously [29]. High-binding-flat bottom 96-well plates (Greiner Bio-one, NC) were coated with either OMPs or flagellin (2 μg/mL for IgG and 7.5 μg/mL for IgA) diluted in 0.05 M sodium -bicarbonate coating buffer (9.6 pH), and incubated overnight at 4˚C. Plates were washed three times with PBS- Tween 20 (PBST) (0.05% Tween 20 in PBS, pH 7.4) and blocked with 5% non-fat dry milk powder in PBST for 1h at room temperature. Plates were washed three times with PBST. For analysis, 50 μl of serum and bile samples were diluted in 2.5% non-fat dry milk, and 50 μl of cloacal supernatants were added to the wells in triplicates. Samples were incubated for 2h at room temperature. Plates were washed three times with PBST. HRP-conjugated goat anti-chicken IgG (Southern Biotech, AL) (1: 10,000 in 2.5% non-fat dry milk powder in PBST) or HRP-conjugated goat anti-chicken IgA (Gallus immunotech, NC) (1: 3000 in 2.5% skim milk powder in PBST) secondary antibodies (50 μL/well) were added and incubated for 2h at room temperature. Plates were washed three times, and 50 μl/well of TMB peroxidase substrate was added. The reaction was stopped after 6 min by adding 2M sulfuric acid. The OD was measured at 450 nm using Gen5TM software (BioTek,VT,USA). IgG and IgA values were reported as the mean optical density. The corrected OD was obtained by subtracting the treatment group OD from blank control OD.

### Cytokine gene expression in the cecal tonsils, liver, and spleen of CNP-vaccine administered birds

Total RNA was extracted using TRI reagent (Molecular Research Center, Cincinnati, OH) following the manufacturer’s instructions. The RNA was reverse-transcribed into cDNA and analyzed for IL-1β, IFNγ, IL-10, and IL-4, mRNA by real-time PCR (CFX96 Touch Real Time System, BioRad) using SyBr green after normalizing for RPS13 and GAPDH. Fold change from the reference was calculated, as explained previously [30]. Primers sequences are described in Table 1.

**Table 1:** Real-time PCR primers for cytokine analysis.

	Primer	Sequence (5’– 3’)	T_a_
GAPDH	F	TCCTGTGACTTCAATGGTGA	55.0°C
	R	CACAACACGGTTGCTGTATC	
RPS13	F	CAAGAAGGCTGTTGCTGTTCG	55.5°C
	R	GGCAGAAGCTGTCGATGATT	
IL-1β	F	TCCTCCAGCCAGAAAGTGA	57.0°C
	R	CAGGCGGTAGAAGATGAAGC	
IL-4	F	AACATGCGTCAGCTCCTGAAT	57.5°C
	R	TCTGCTAGGAACTTCTCCATTGAA	
IFNγ	F	GTGAAGAAGGTGAAAGATATCATGGA	57.0°C
	R	GCTTTGCGCTGGATTCTCA	
IL-10	F	GAGGAGCAAAGCCATCAAGC	57.5°C
	R	CTCCTCATCAGCAGGTACTCC	

Primers were adapted from previous literature. Glyceraldehyde 3-phosphate dehydrogenase (GAPDH), Dube et al. 2014 [31]; Ribosomal protein S13 (RPS13), Shanmugasundaram et al. 2018; IL-1β, IFNγ, and IL-10, Shanmugasundaram et al. 2019 [32]; IL-4, Renu et al. 2018 [29].

### Experiment II

Experiment II studied the cross-protective effect of synthesized CNP vaccine against *S*. Heidelberg infection. A total of 216 Cobb-500 one-day-old chicks were randomly assigned to six treatments. Each treatment groups had 36 birds in 6 pens (n = 6) with 6 birds/pen. The six treatments involved in the study were distributed as a 3 (No Vaccine, CNP vaccine and Commercial vaccine) X 2 (*S*. Enteritidis and *S*. Heidelberg challenge) factorial set up of treatments. CNP vaccine groups received 1000μg of CNP vaccine in 100μL of PBS at d1 followed by the same dose as a booster dose on d7 orally. Commercial vaccine group received 100 μl of live *Salmonell*a vaccine (Poulvac ST, Zoetis, NJ) at d1 followed by the same dose as a booster dose on d7 orally. The birds in the control group received 100μl of PBS orally. *S*. Enteritidis or *S*. Heidelberg challenge treatments were conducted in two different rooms to avoid cross-infection. Birds had access to *ad libitum* feed and water.

At d1, 7, 14, and 18 body weight and feed consumption were recorded, and body weight gain and feed consumption ratio was calculated. At 14d of age, birds were challenged with an oral gavage with 1 X 10^5^ CFU/bird of live *S*. Enteritidis or *S*. Heidelberg. At d1, 7, 14 (8h-post-challenge), 16, and 18 of age, serum was collected to analyze anti-OMPs and anti-flagellin IgG. At d1, 7, 14 (8h-post-challenge), 16, and 18 of age, cloacal swab samples were collected to analyze anti-OMPs and anti-flagellin IgA. At d18 (4d-post-challenge) of age, bile samples were collected to analyze anti-OMPs and anti-flagellin IgA. At d18 (4 post-challenge) cecal tonsils were collected and analyzed for IL-1β and IL-10 cytokine mRNA amounts. At d16 and 18 (2 and 4 post-challenge), cecal content liver and spleen were collected and analyzed for *Salmonella* loads.

### *Salmonella* loads in the ceca of CNP-vaccine administered birds

Cecal content was collected from one bird from each pen (n = 6) and analyzed for *S*. Enteritidis loads by real time PCR. Bacterial genomic DNA was isolated as described earlier [30]. Cecal samples (200 mg) were washed two times with 1X PBS. The cell pellet was resuspended in EDTA and treated with 20 mg/ml lysozyme for 30 min at 37°C. The samples were treated with lysis buffer containing 20% SDS and 0.1 mg/ml proteinase K (Sigma Aldrich, St Louis, MO) for 5 min at -80°C. The samples were incubated with 5μL of RNase at 37°C for 30 min. The cell lysate was incubated with 6M sodium chloride on ice for 10 min. The supernatant was collected after centrifugation at 400 X g for 10 min. The DNA in the supernatant was precipitated with isopropanol and washed once in ice-cold ethanol. The DNA pellet was resuspended in TE buffer (10 mM Tris-HCl, 1 mM EDTA, pH 8.0) and stored at -20°C until further use.

The DNA extracted from the different treatment groups was analyzed for *S*. Enteritidis loads by real-time PCR using *Salmonella Enteritidis* primers 5’-GCAGCGGTTACTATTGCAGC-3’ and 5’-CTGTGACAGGGACATTTAGCG-3 [33]. The threshold cycle (Ct) values were determined by iQ5 software (Bio-Rad, Hercules, CA) when the fluorescence rises exponentially 2-fold above background. The copy numbers of *S*. Enteritidis was expressed in log units as described earlier [34].

### *Salmonella* loads in the liver and spleen of CNP-vaccine administered birds

*Salmonella* loads in the liver and spleen samples was determined by a 3-tube Most Probable Number (MPN) method. The samples were homogenized in 1: 2 (W/V) of buffered peptone water (BPW). Samples were prepared in 10-fold dilution series, and then 1mL of each dilution was inoculated into triplicate broth culture tubes for incubation at 42°C for 24 h. Following incubation, all tubes are examined for turbidity, and the pattern of growth in the tubes was scored against an MPN table from the U.S. Food and Drug Administration’s Bacterial Analytical Manual [35]. The bacteria in the broth were confirmed to be *Salmonella* by plating one hundred microliters of each positive culture tube onto XLT4 Agar plates (25 mg/mL novobiocin) and incubated for 24h at 42°C for confirmation of black colonies and MPN index/g. Samples were bacterial growth and had no black colonies on the XLT4 agar plates were discarded as false positives and assigned an MPN value of 0.

#### Statistical analyses

All data in Experiment I and II were examined by one-way ANOVA (JMP, SAS Institute Inc., Cary, NC), to examine the effect of CNP-*Salmonella* vaccine during *S*. Enteritidis and *S*. Heidelberg. When the treatment effects were significant (*P<*0.05) differences between means were analyzed using Tukey’s test. MPN-non-parametric data was transformed to Log10 before statistical analysis.

## Results and discussion

### Effect of CNP vaccine on bird production performance

In both experiments, there were no significant differences in body weight gain and feed conversion ratio of birds in different treatment groups indicating that CNP vaccine administration had no adverse effects on broilers’ performance parameters (S1 Table).

### Effect of CNP vaccine on macrophage-nitrite production

**Fig 1:**
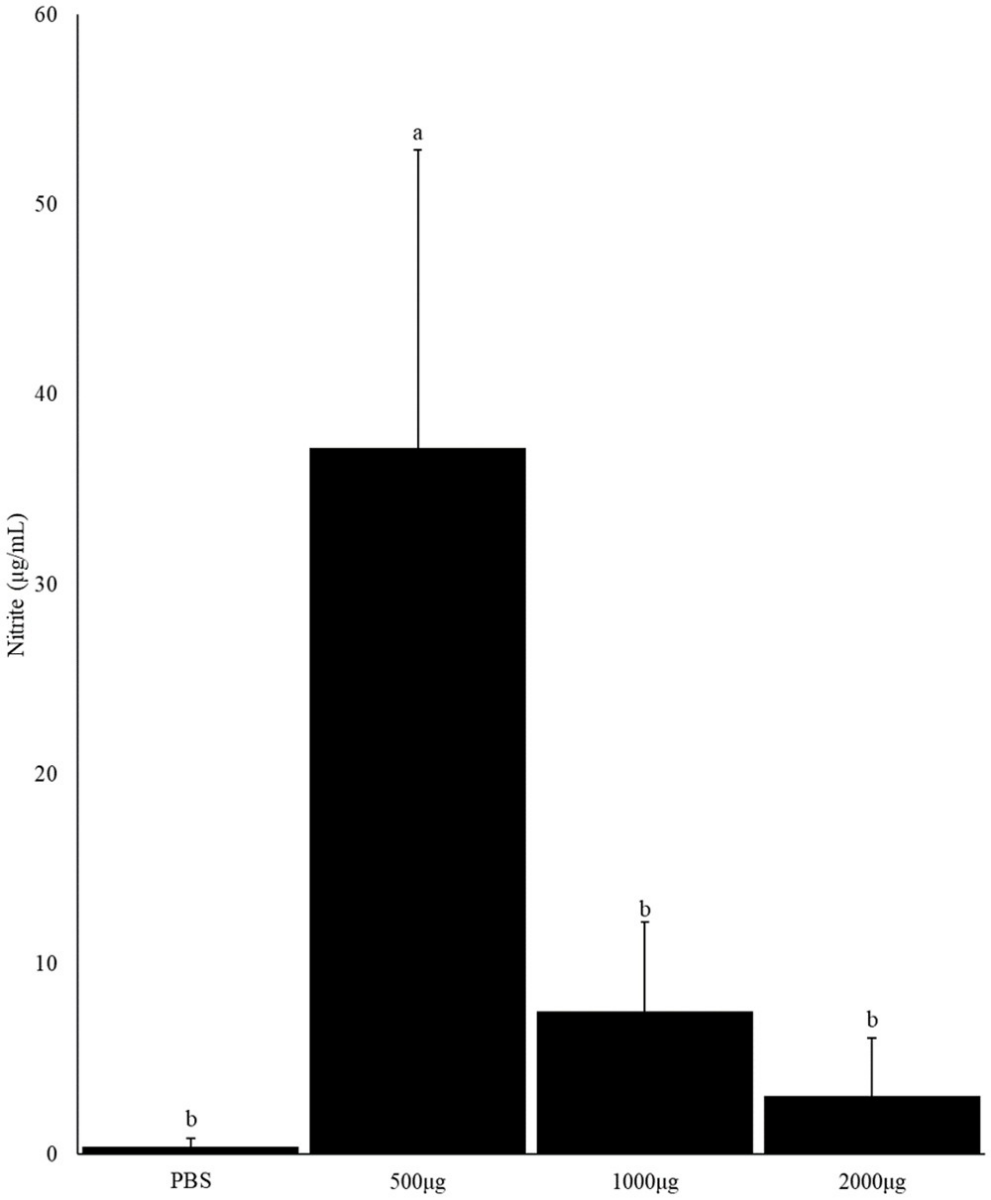
Effect of CNP–*Salmonella* vaccine on macrophage-nitrite production post-*S*. Enteritidis challenge. At 1d of age, chickens were orally vaccinated with either PBS (control-challenged) or different doses of outer membrane + flagellin proteins: 500μg, 1000 μg, or 2000 μg, loaded into CNP. The same route of delivery and doses was repeated at d7 of age. At d14 of age, birds were challenged with live *S*. Enteritidis (5.4 x 10^5^ CFU/bird). Splenic macrophages were isolated from broilers at d25 (11d-post-challenge) of age and cells were stimulated with 1 μg/mL *S*. Enteritidis LPS. The nitrite content of the supernatant was determined using Griess assay. Results were reported as average optical density (OD) values. Bars (+SE) with no common superscript differ (*P<*0.05). n = 3 (Exp 1).

In Experiment I, birds vaccinated with 500μg CNP vaccine had higher levels (*P<*0.05) of nitrite compared to PBS treatment group (Fig 1). Birds vaccinated with 1000μg, and 2000μg CNP vaccine had non-significant higher levels of nitrite compared to PBS treatment group. Within the CNP vaccine administered group, increasing the CNP vaccine dose from 500 to 2000μg decreased the macrophage-nitrite content. Previous *in vitro* studies have identified that *S*. Enteritidis infection suppresses nitirite production in chicken macrophage HD11-cells [36], which can be expected to facilitate the pathogen escapting the host immune response. However, inflammatory responses, such as nitric oxide production, can decrease production performance in broilers [37]. Birds adminstered with 1000μg CNP vaccine had the highest amount of nitirte suggestingt that 1000μg dose can provide protection against *Salmonella* challenge.

### Effect of CNP vaccine administration on anti-OMPs and–flagellin IgG and IgA antibody response

**Fig 2:**
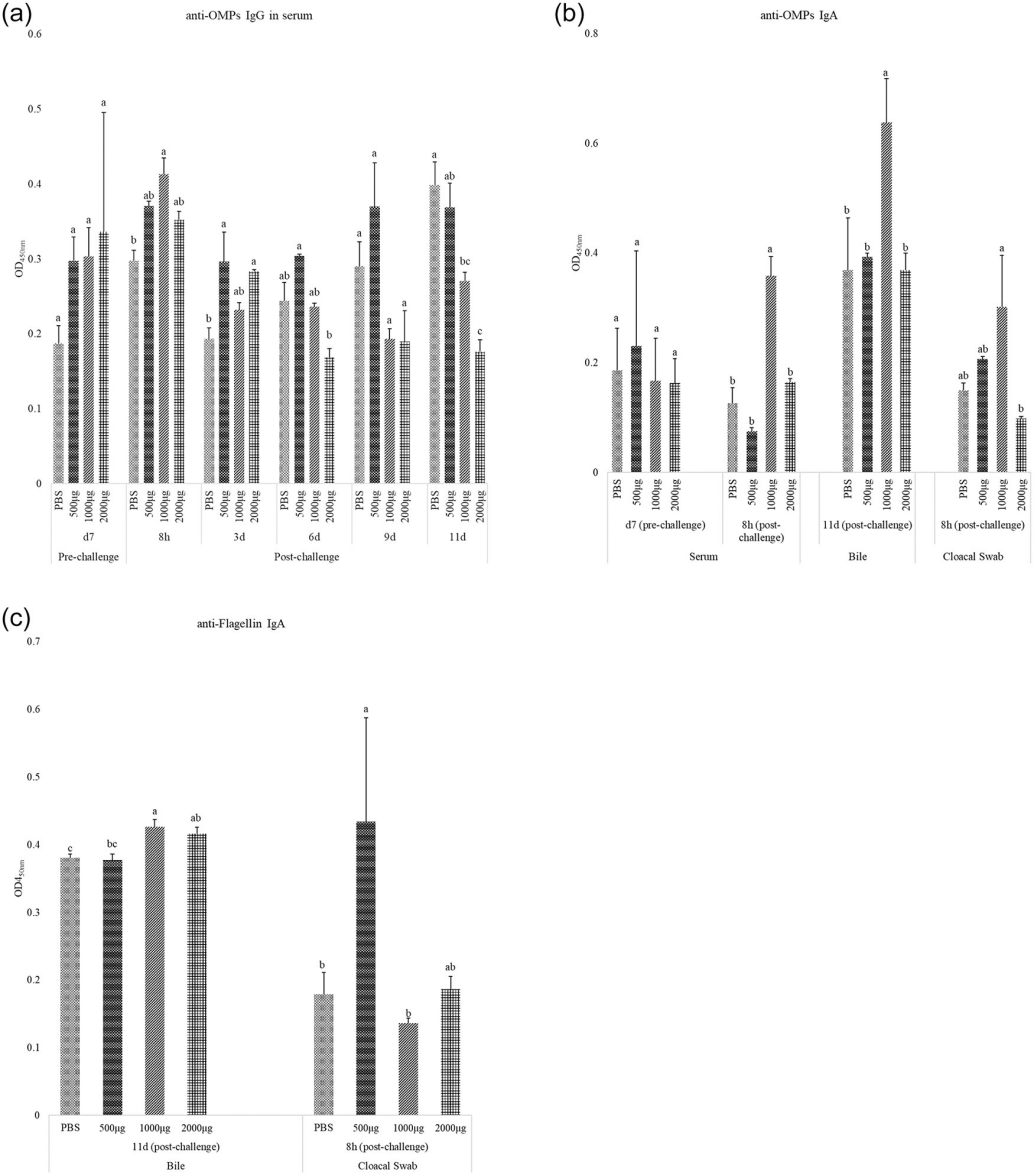
Effect of CNP-*S*. Enteritidis vaccine on anti-*Salmonella* IgG and IgA antibody levels. At 1d of age, chickens were orally vaccinated with either PBS (control-challenged) or different doses of outer membrane + flagellin proteins: 500μg, 1000 μg, or 2000 μg, loaded into CNP. The same route of delivery and doses was repeated at d7 of age. At d14 of age, birds were challenged with live *S*. Enteritidis (5.4 x 10^5^ CFU/bird). Blood, bile and cloacal swab samples were collected pre- and post-challenge and analyzed for anti-*Salmonella* antigen-specific IgG and IgA levels using ELISA. Results were reported as average optical density (OD) values. A–OMPs IgG; B–OMPs IgA; C–Flagellin IgA. Bars (+ SE) with no common superscript differ (P<0.05). n = 3 to 5.

In Experiment I, at 8h- post-challenge broilers that were vaccinated with 1000μg CNP dose and orally challenged with *S*. Enteritidis had 39% higher (*P<*0.05) serum anti-OMP IgG compared to that in the control group (Fig 2A). At 3d-post-challenge, birds vaccinated with 500μg and 2000μg CNP vaccine dose had higher (*P<*0.05) serum anti-OMP IgG compared to that in the control group (Fig 2A). These results demonstrated that the CNP vaccine at doses above 500μg can induce an anti-*Salmonella* antigen-specific immune response in broilers. Similar results were observed in a recent study, where layer chickens that were orally immunized with a *Salmonella* polyanhydride nanoparticle (PNP) vaccine and challenged with *S*. Enteritidis had substantially higher OMPs-specific IgG response in the serum [29].

At 8h-post-challenge, birds vaccinated with 1000μg CNP had 185% higher (*P<*0.05) anti- OMPs serum IgA amounts compared to that in the control-challenge groups (Fig 2B). At 8h-post-challenge, birds vaccinated with 500μg and 2000μg *Salmonella*-CNP vaccine had similar serum anti-OMPs IgA levels. At 11d-post-challenge, birds vaccinated with 1000μg CNP had 73% higher bile anti-OMPs IgA (Fig 2B) and 12% higher anti-flagellin IgA (Fig 2C), compared to that from the control group (*P<*0.05). At 8h-post-challenge, birds vaccinated with 1000μg CNP had 208% higher cloacal swabs anti- OMPs IgA (*P<*0.05), compared to that from the control group (Fig 2B). However, at 8h-post-challenge birds vaccinated with 500μg CNP had higher cloacal swabs anti-*S*. Enteritidis flagellin IgA (*P<*0.05), compared to that from the control (Fig 2C). At d25 (11d-post-challenge), birds vaccinated with 2000μg CNP had lower serum anti-OMPs IgG (*P<*0.05), compared to that from the control group (Fig 2A).

Experiment I results identified that 1000μg CNP vaccine induced *Salmonella* antigen-specific immune response against *S*. Enteritidis in broilers and hence 1000μg CNP vaccine dose was further studied in Experiment II. f

**Fig 3:**
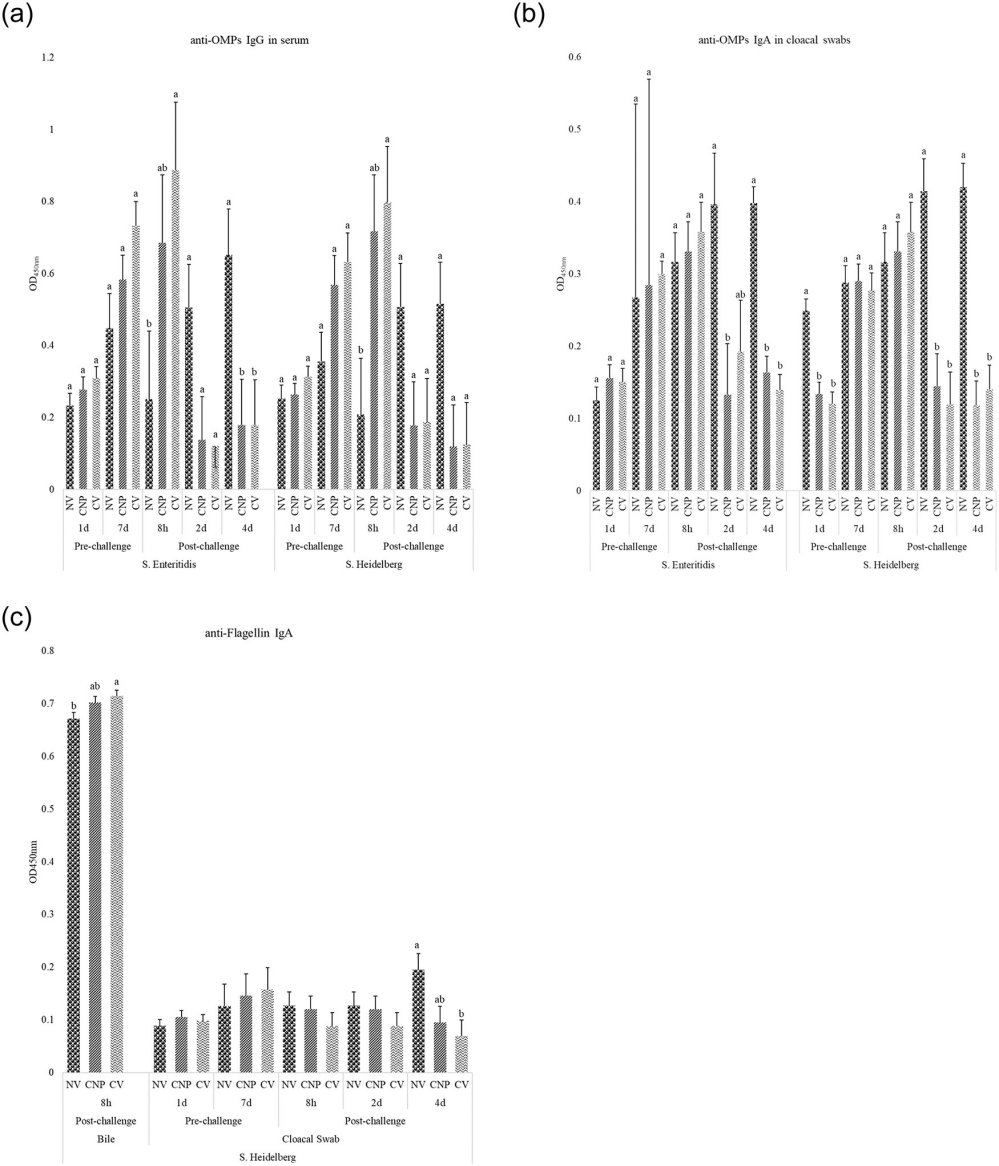
Effect of CNP-*Salmonella* vaccine on anti-*S*. Enteritidis or Heidelberg IgG and IgA antibody levels. At 1d of age, chickens were orally vaccinated with either PBS (NV), 1000 μg chitosan nanoparticle vaccine (CNP) or a commercial vaccine (CV). The same route of delivery and doses was repeated at 7d of age. At 14d of age, birds were challenged with 1 X 10^5^ CFU/bird of either *S*. Enteritidis or *S*. Heidelberg. Blood, bile and cloacal swab samples were collected pre- and post-challenge and analyzed for anti-*Salmonella* antigen-specific IgG and IgA levels using ELISA. Results were reported as average optical density (OD) values. A–OMPs IgG; B–OMPs IgA; C–Flagellin IgA. Bars (+ SE) with no common superscript differ (P<0.05). n = 6.

In Experiment II, at 8h-post-challenge, birds vaccinated with the commercial vaccine or the CNP had 253% and 173% higher (*P<*0.05) anti-OMPs serum IgG amounts, respectively, compared to that in the no-vaccine control (Fig 3A). At 4d-post-challenge, birds vaccinated with the commercial vaccine or the CNP had 72.63% and 72.62% lower (P<0.05) anti-OMPs serum IgG amounts, respectively, compared to that in the control (Fig 3A) group. These results identified that vaccinating birds with the CNP vaccine induces a high titer of *Salmonella* specific protective antibodies and in the absence of infection, the antibody titers will eventually decline over time [38]. In contrast, a carrier animal will usually maintain persistently high antibody levels in the blood, indicative of a continuing infection [39], as previously documented with *S*. Dublin in cattle [39]. Similarly, at 2d-post-*S*. Enteritidis challenge, birds vaccinated with the commercial vaccine or CNP vaccine had 51.42% and 66.5% lower (*P<*0.05) cloacal swabs anti- OMPs IgA amounts, respectively, compared to that in the control group (Fig 3B). At 4d-post-*S*. Enteritidis challenge, birds vaccinated with the commercial vaccine or CNP vaccine had 65.13% and 59% lower (*P<*0.05) cloacal swabs anti-OMPs IgA amounts, respectively, compared to that in the control (Fig 3B).

Results for *S*. Heidelberg showed that at d7, birds vaccinated with commercial vaccine or CNP had 51.7% and 46.3% lower (*P<*0.05) cloacal anti-*S*. Heidelberg OMPs IgA amounts, respectively, compared to that in the control group (Fig 3B). However, after adminstrating the booseter dose, there was no differences in the cloacal anti-OMPs IgA amounts among all treatment groups (Fig 3B). A possible explanation for this could be a) the transfer of maternal antibodies [40] or b) pre-existing *Salmonella* colonization from exposure to exogenous microbes either in the hatchery or farm environment [41]. It has been reported that within broiler hatcheries, 74% of pad samples placed under newly hatched chicks were positive for *Salmonella* [42].

At 2d-post-*S*. Heidelberg challenge, birds vaccinated with the commercial vaccine or CNP vaccine had 71.2% and 65.1% lower (P<0.05) cloacal anti-*S*. Heidelberg OMPs IgA amounts, respectively, compared to that in the control group (Fig 3B). At 4d-post-*S*. Heidelberg challenge, birds vaccinated with the commerical vaccine or the CNP vaccine had 66.3% and 71.8% lower (P<0.05) cloacal anti-*S*. Heidelberg OMPs IgA amounts, respectively, compared to that in the control group (Fig 3B). At 4d-post-*S*. Heidelberg challenge, birds vaccinated with the commerical vaccine or CNP vaccine had 64.36% and 51.2% lower (*P<*0.05) cloacal anti-*S*. Heidelberg flagellin IgA amounts, respectively, compared to that in the control group (Fig 3C). In addition, at 8h-post-*S*. Heidelberg challenge, birds vaccinated with the commerical vaccine or CNP vaccine had 6.36% and 4.59% higher (*P<0*.05) bile anti-*S*. Heidelberg flagellin IgA amounts, respectively, compared to that in the control group (Fig 3C). At 8h-post-*S*. Heidelberg challenge, birds vaccinated with the commerical vaccine or CNP vaccine had 253% and 173% higher (*P<0*.05) serum anti-*S*. Heidelberg OMPs IgG amounts, respectively, compared to that in the control group (Fig 3A). These results demonstrate that CNP can elicit an antigen-specfic and cross-protective immune response against *S*. Heidelberg in broilers.

### Effect of CNP vaccine on *S*. Enteritidis loads in ceca at 2d-post-challenge

*Salmonella* Enteritidis frequently colonizes the GIT of poultry [2]; hence *Salmonella* loads in cecal contents was quantified by real-time qPCR. In Experiment I, there were no significant differences in *S*. Enteritidis loads in cecal content of birds in different treatment groups at d25 (11d-post-challenge) (*P>*0.05).

In Experiment II, *S*. Heidelberg and *S*. Enteritidis loads in liver and spleen was analyzed using MPN method while *S*. Heidelberg and *S*. Enteritidis loads in the cecal content was analyzed by real-time qPCR. At 4d post-challenge, there were no significant differences (*P>*0.05) in *S*. Heidelberg loads in the liver, spleen (Tables 2 and 3) or ceca among birds in different treatment groups. However, *S*. Heidelberg loads in liver and spleen was numerically lower in birds vaccianted with commerical vaccine and CNP vaccine compared to that in the control group.

**Table 2:** *S*. Heidelberg loads in liver of birds in different treatment groups at 4d-post-*S*. Heidelberg challenge.

Sample	100 μl	10 μl	1 μl	Colonies counted (10^−5^)	MPN index/g	Log10	Average MPN value	SEM
Control	2	1	0	0	0	0	0.57	0.36
	3	0	2	63	64	1.8		
	3	0	0	0	0	0		
	2	2	1	0	0	0		
	2	2	2	0	0	0		
	3	0	1	37	38	1.6		
CNP	1	2	1	13	15	1.2	0.20	0.20
	2	0	0	0	0	0		
	1	0	0	0	0	0		
	1	1	0	0	0	0		
	2	0	2	0	0	0		
	3	0	0	0	0	0		
Commercial Vaccine	1	1	0	0	0	0	0.22	0.22
	0	1	0	0	0	0		
	1	1	0	0	0	0		
	1	0	0	0	0	0		
	1	0	0	0	0	0		
	2	0	2	20	20	1.3		
*P-*value	*P>*0.05							

At 1d of age, chickens were orally vaccinated with either PBS (NV), 1000 μg chitosan nanoparticle vaccine (CNP) or a commercial vaccine (CV). The same route of delivery and doses was repeated at 7d of age. At 14d of age, birds were challenged with 1 X 10^5^ CFU/bird of either *S*. Enteritidis or *S*. Heidelberg. At 4d-post-challenge *Salmonella* loads in the liver samples was determined by the MPN method. Samples with no bacterial growth were assigned an MPN of 0. Chitosan Nanoparticle (CNP). n = 6. Means with no common superscript differ (*P<*0.05).

**Table 3:** *S*. Heidelberg loads in spleen of birds in different treatment groups at 4d-post-*S*. Heidelberg challenge.

Sample	100 μl	10 μl	1 μl	Colonies counted (10^−5^)	MPN index/g	Log10	Average MPN value	SEM
Control	3	3	1	0	0	0	1.15	0.38
	3	1	2	25	120	2.1		
	3	1	1	4	75	1.9		
	2	2	2	15	35	1.5		
	2	2	2	0	0	0		
	3	0	0	14	23	1.4		
CNP	3	2	1	9	150	2.2	0.58	0.39
	2	2	0	4	21	1.3		
	1	0	0	0	0	0		
	3	2	1	0	0	0		
	2	2	2	0	0	0		
	2	0	0	0	0	0		
Commericial Vaccine	1	1	1	2	11	1.0	0.57	0.40
	2	1	0	0	0	0		
	1	1	0	0	0	0		
	1	0	0	0	0	0		
	3	3	0	12	240	2.4		
	2	0	0	0	0	0		
*P-*value	*P>*0.05							

At 1d of age, chickens were orally vaccinated with either PBS (NV), 1000 μg chitosan nanoparticle vaccine (CNP) or a commercial vaccine (CV). The same route of delivery and doses was repeated at 7d of age. At 14d of age, birds were challenged with 1 X 10^5^ CFU/bird of either *S*. Enteritidis or *S*. Heidelberg. At 4d-post-challenge *S*. Heidelberg loads in the spleen samples was determined by the MPN method. Samples with no bacterial growth were assigned an MPN of 0. Chitosan Nanoparticle (CNP). n = 6. Means with no common superscript differ (*P<*0.05).

**Fig 4:**
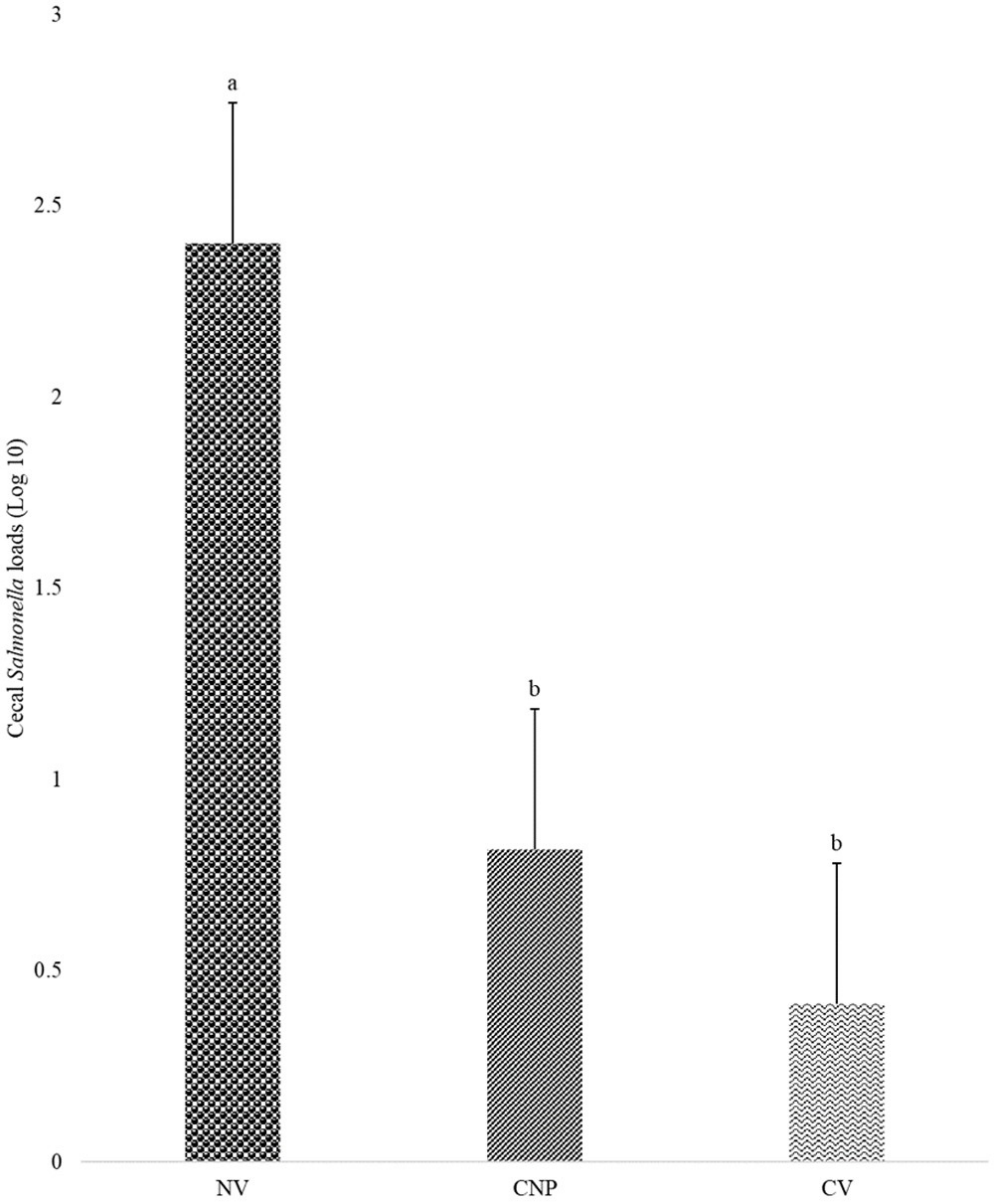
Quantification of *S*. Enteritidis on cecal content colonization at 2d- post-challenge. At 1d of age, chickens were orally vaccinated with either PBS (NV), 1000 μg chitosan nanoparticle vaccine (CNP) or a commercial vaccine (CV). The same route of delivery and doses was repeated at 7d of age. At 14d of age, birds were challenged with 1 X 10^5^ CFU/bird of either *S*. Enteritidis or *S*. Heidelberg. At 4d-post-challenge *S*. Enteritidis loads in the cecal content was determined by real-time PCR. The copy numbers of *S*. Enteritidis was expressed in log units. Bars (+SE) with no common superscript differ (*P<*0.05). No Vaccine (NV); Chitosan Nanoparticle Vaccine (CNP); Commercial Vaccine (CV). n = 6.

In Experiment II, at d18 (4d-post-challenge) there was no *S*. Enteritidis colonization in the liver and spleen. At d16 (2d-post-challenge) birds vaccinated with commerical vaccine or CNP vaccine had lower (*P<*0.05) *Salmonella* load by 82.7% and 65.9%, respectively, when compared to that in the control group (Fig 4). Similar results were observed in a recent 2018 study, as a PNP vaccination cleared *Salmonella* in the ceca of 33% of birds in the study [29]. This identified that the synthesized vaccine decreased *Samonell*a loads in the ceca of broilers.

### Effect of CNP vaccine on cytokine gene expression by qPCR

**Fig 5:**
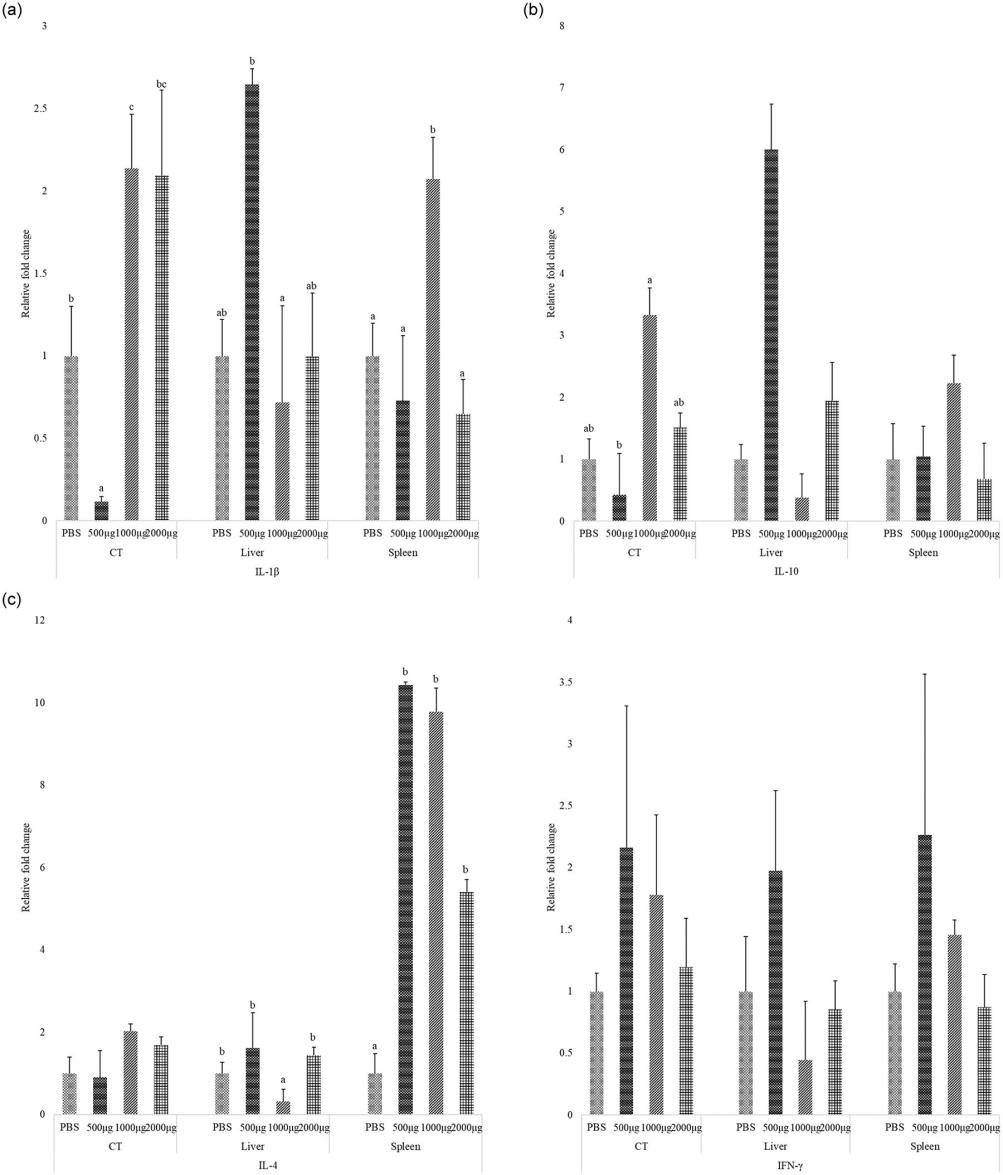
Effect of CNP vaccine on cytokine gene expression by qPCR. At 1d of age, chickens were orally vaccinated with either PBS (control-challenged) or different doses of outer membrane + flagellin proteins: 500μg, 1000 μg, or 2000 μg, loaded into CNP. The same route of delivery and doses was repeated at d7 of age. At d14 of age, birds were challenged with live *S*. Enteritidis (5.4 x 10^5^ CFU/bird). Cecal tonsil, liver and spleen samples were collected at 11d-post-challenge and analyzed for cytokine mRNA amounts. A–IL-1β mRNA; B–IL-10 mRNA; C–IL-4 mRNA; D–IFNγ mRNA. Bars (+SEM) with no common superscript differ (P<0.05). n = 6.

The effect of CNP vaccine on four key cytokines that influence both innate and adaptive immune responses, IL-1β (Fig 5A), IL-10 (Fig 5B), IL-4 (Fig 5C), and IFNγ (Fig 5D) were studied. In Experiment I, liver and spleen samples were analyzed to identify any adverse effects of CNP vaccine on liver and spleen, as *S*. Enteritidis colonizes in liver and spleen at 1d post- inoculation [43,44].

In Experiment I, at d25 (11d-post-challenge), birds immunized with 500μg, 1000μg or 2000μg CNP had no effect on cecal tonsil IL-4 and IFNγ mRNA amounts (*P*>0.05), but had higher IL-1β mRNA amounts in cecal tonsils, spleen, and liver samples (*P<*0.05). Cecal tonsil IL-1β mRNA amount was 10-fold lower (*P<*0.05) in the 500μg CNP-vaccinated birds while the 1000μg and 2000μg CNP vaccinated birds had two-fold higher (*P<*0.05) IL-1β, compared to that in the control group. Spleen IL-1β mRNA amount was two-fold higher (*P<*0.05) in the 1000μg CNP-vaccinated birds, compared to that in the control group. Liver IL-1β mRNA amount in the 500μg CNP-vaccinated group was 2.6-fold higher (*P<*0.05), compared to that in the control group, while the 1000μg-vaccinated birds had 0.7-fold lower (*P<*0.05) IL-1β mRNA amounts compared to that in the control group. A possible explanation for increased mRNA content of pro-inflammatory cytokine IL-1β is that the adjuvant composition of the vaccine can induce a predominantly Th1 type inflammatory response [26]. The only vaccine dose that increased the anti-inflammatory cytokine IL-10 amounts was the 1000μg CNP vaccine. Cecal tonsil IL-10 mRNA content was two-fold lower in the 500μg CNP-vaccinated birds, while cecal tonsil IL-10 mRNA content was 3.3-fold higher (*P<*0.05) in the 1000μg CNP treatment groups, compared to that in the control group. IL-10 cytokine reduces host tissue damage in response to inflammation caused by bacterial infections [45] and hence 1000μg CNP vaccine dose was chosen for Experiment II.

Results show that birds vaccinated with 500μg, 1000μg or 2000μg CNP had higher spleen IL-4 mRNA amounts, compared to that of the control (*P<*0.05). Liver IL-4 mRNA amounts were three-fold lower in the 1000μg vaccinated group, compared to that in the control group (*P<*0.05). IL-4, a Th2 cytokine, can decrease the production of IFNγ cytokine and decrease the activation of macrophages [38]. However, no significant differences were observed on IFNγ mRNA content among birds in different treatment groups. At d25 (11d-post-challenge), birds vaccinated with 500μg, 1000μg or 2000μg CNP had no differences in spleen and liver IL-10 and IFNγ mRNA amounts (*P>*0.05), compared to that in the control groups. In Experiment II, at d18 (4d-post-*S*. Heidelberg or *S*. Enteritidis challenge) there were no significant differences in cecal tonsil IL-1β or IL-10 mRNA amounts (*P>*0.05). Results from Experiment I and II demonstrated that the CNP vaccine had no adverse effects on the bird’s health.

## Conclusion

The vaccine under study has previously shown to induce substantially higher antigen-specific IgA response in bile, serum, cloacal swab, and tracheal wash samples of layer birds [26]. This study identified that the 1000μg CNP-*Salmonell*a vaccine can substantially increase antigen-specific anti-*Salmonella* IgG and IgA amounts in broilers infected with either *S*. Enteritidis or *S*. Heidelberg, providing cross-protection against both *Salmonella* enterica serovars. The 1000μg CNP vaccine did not affect the production performance of birds and induced substantial IL-1β and IL-10 cytokines, and nitrite productuon in response to *S*. Enteritidis infection, thereby identifying the CNP vaccine as a potential vaccine candidate. The 1000μg CNP vaccine significantly decreased the cecal colonization of *S*. Enteritidis as well as *S*. Heidelberg in vacinated broilers, idnetifying the CNP vaccine as a potential candicate that can mitigate *Salmonella* loads. Results show that the CNP vaccine is a potential vaccine candidate that can be deliveed orally to control *Salmonella* infections in poultry.

## Supporting information

S1 Table Effect of CNP vaccine on performance parameters in *Salmonella* challenged birds. A) Final body weight gain (BWG) and feed consumption ratio (FCR) of d25 broilers in Experiment I. At 1d and 7d of age, chickens were orally vaccinated with 0.1 mL of PBS (control-challenge) or 500μg, 1000μg, or 2000μg CNP vaccine. At 14d of age, birds were challenged using an oral gavage with 1 X 10^5^ CFU/bird of *S*. Enteritidis. BWG and FCR was calculated on d25 of age. B) Final BWG and FCR of d18 broilers in Experiment II—At 1d and 7d of age, chickens were orally vaccinated with 0.1 mL of PBS (control-challenge) or 1000μg CNP vaccine, or a live commercial *Salmonell*a vaccine at d1 and d7. At 14d of age, birds were challenged using an oral gavage with 1 X 10^5^ CFU/bird of either live *S*. Enteritidis or live *S*. Heidelberg. BWG and FCR was calculated on d18 of age. n = 6. Means (SEM) with no common superscript differ (*P<*0.05).
(PDF)
