# Supplementary material for: Efficacy of chitosan-based nanoparticle vaccine administered to broiler birds challenged with Salmonella
Source: PLoS One. 2020 Apr 24;15(4):e0231998. doi: 10.1371/journal.pone.0231998 (PMC7182187; doi:10.1371/journal.pone.0231998)
Supplement: S1 Table — A) Final body weight gain (BWG) and feed consumption ratio (FCR) of d25 broilers in Experiment I. At 1d and 7d of age, chickens were orally vaccinated with 0.1 mL of PBS (control-challenge) or 500μg, 1000μg, or 2000μg CNP vaccine. At 14d of age, birds were challenged using an oral gavage with 1 X 105 CFU/bird of S. Enteritidis. BWG and FCR was calculated on d25 of age. B) Final BWG and FCR of d18 broilers in Experiment II—At 1d and 7d of age, chickens were orally vaccinated with 0.1 mL of PBS (control-challenge) or 1000μg CNP vaccine, or a live commercial Salmonella vaccine at d1 and d7. At 14d of age, birds were challenged using an oral gavage with 1 X 105 CFU/bird of either live S. Enteritidis or live S. Heidelberg. BWG and FCR was calculated on d18 of age. n = 6. Means (SEM) with no common superscript differ (P<0.05). (PDF) [file pone.0231998.s001.pdf]

A. Experiment 1 - Final (d25) BWG & FCR				
**Parameter**	**BWG (g)**	**SEM**	**FCR**	**SEM**
**Control (PBS)**	155.46	45.35	1.82	0.21
**500µg**	137.37	45.35	1.30	0.10
**1000µg**	150.12	26.98	1.37	0.15
**2000µg**	153.09	26.52	1.30	0.21
***P*-value**	$P>0.05$			
**B. Experiment 2 - Final (d18) BWG & FCR**				
***S.* Heidelberg challenge**	**BWG (g)**	**SEM**	**FCR**	**SEM**
**Control (no vaccine)**	415.46	47.35	1.20	0.14
**CNP vaccine**	450.37	45.02	1.30	0.15
**Commercial vaccine**	455.12	26.98	1.37	0.11
***P*-value**	$P>0.05$			
***S.* Enteritidis challenge**	**BWG (g)**	**SEM**	**FCR**	**SEM**
**Control (no vaccine)**	423.46	45.35	1.25	0.14
**CNP vaccine**	415.37	43.05	1.14	0.15
**Commercial vaccine**	420.12	26.98	1.16	0.11
***P*-value**	$P>0.05$			
